# Alterations of gene expression and protein synthesis in co-cultured adipose tissue-derived stem cells and squamous cell-carcinoma cells: consequences for clinical applications

**DOI:** 10.1186/scrt454

**Published:** 2014-05-12

**Authors:** Eva Koellensperger, Felix Gramley, Fabian Preisner, Uwe Leimer, Guenter Germann, Verena Dexheimer

**Affiliations:** 1 Clinic for Plastic and Reconstructive Surgery, Aesthetic and Preventive Medicine at Heidelberg University Hospital – ETHIANUM, Voßstraße 6, 69115 Heidelberg, Germany; 2 Department of Cardiology, University of Frankfurt, Theodor-Stern-Kai 7, 60590 Frankfurt, Germany

## Abstract

**Introduction:**

This is the first study evaluating the interactions of human adipose tissue derived stem cells (ADSCs) and human squamous cell carcinoma cells (SCCs), with regard to a prospective cell-based skin regenerative therapy and a thereby unintended co-localization of ADSCs and SCCs.

**Methods:**

ADSCs were co-cultured with A431-SCCs and primary SCCs (pSCCs) in a transwell system, and cell-cell interactions were analyzed by assessing doubling time, migration and invasion, angiogenesis, quantitative real time PCR of 229 tumor associated genes, and multiplex protein assays of 20 chemokines and growth factors and eight matrix metalloproteinases (MMPS). Results of co-culture were compared to those of the respective mono-culture.

**Results:**

ADSCs’ proliferation on the plate was significantly increased when co-cultured with A431-SCCs (*P* = 0.038). PSCCs and ADSCs significantly decreased their proliferation in co-culture if cultured on the plate (*P* <0.001 and *P* = 0.03). The migration of pSCC was significantly increased in co-culture (*P* = 0.009), as well as that of ADSCs in A431-SCC-co-culture (*P* = 0.012). The invasive behavior of pSCCs and A431-SCCs was significantly increased in co-culture by a mean of 33% and 35%, respectively (*P* = 0.038 and *P* <0.001). Furthermore, conditioned media from co-cultured ADSC-A431-SCCs and co-cultured ADSCs-pSCCs induced tube formation in an angiogenesis assay *in vitro*.

In A431-SCC-co-culture 36 genes were up- and 6 were down-regulated in ADSCs, in A431-SCCs 14 genes were up- and 8 genes were down-regulated. In pSCCs-co-culture 36 genes were up-regulated in ADSCs, two were down-regulated, one gene was up-regulated in pSCC, and three genes were down-regulated. Protein expression analysis revealed that three proteins were exclusively produced in co-culture (CXCL9, IL-1b, and MMP-7). In A431-SCC-co-culture the concentration of 17 proteins was significantly increased compared to the ADSCs mono-culture (2.8- to 357-fold), and 15 proteins were expressed more highly (2.8- to 1,527-fold) compared to the A431-SCCs mono-culture. In pSCC-co-culture the concentration of 10 proteins was increased compared to ADSCs-mono-culture (2.5- to 77-fold) and that of 15 proteins was increased compared to pSCC mono-culture (2.6- to 480-fold).

**Conclusions:**

This is the first study evaluating the possible interactions of primary human ADSCs with human SCCs, pointing towards a doubtlessly increased oncological risk, which should not be neglected when considering a clinical use of isolated human ADSCs in skin regenerative therapies.

## Introduction

Skin therapies based on adipose tissue derived stem cells (ADSCs) are becoming more and more attractive, regarding the possible positive effects of mesenchymal stem cells on skin cells, such as an increase in collagen content, an improvement of nutrition by increased angiogenesis, a “whitening” effect through inhibition of tyrosine kinase effect, a reduced apoptosis and a UV-protection of dermal fibroblasts [1-5]. ADSCs have been shown to secrete various growth factors that transmit these effects through a paracrine fashion. This mechanism, however, allows ADSCs to interact with tumor cells, too [6,7]. Cancer-associated fibroblasts (CAFs) have been shown to be a crucial component in tumor cell proliferation, cancer invasion and progression by secreting various growth factors, cytokines and proteases into the tumor microenvironment [8]. Since the origin of CAFs is discussed controversially and bone marrow-derived mesenchymal stem cells have also been suggested to be a potential source of CFAs, the question arises of what would happen if ADSCs were injected within the vicinity of local tumor cells, such as skin tumor cells. While one would avoid purposefully injecting ADSCs in a visible skin tumor, of course, an unintended injection of ADSC adjacent to subclinical skin tumor cells is conceivable.

The squamous cell carcinoma is the second-most common skin tumor with a rising incidence of 25/100,000 people in Europe at the moment [9]. So far squamous cell carcinoma cells (SCCs) have not yet been analyzed in this context. Considering the rising use of fat and stem cell-enriched fat as well as the proposed use of isolated ADSCs, for example, in skin regenerative therapies, it is necessary to analyze the possible interactions of human ADSC with human squamous cell carcinoma cells.

To gain further insight in respect to such interactions, we co-cultured primary human ADSCs and primary SCCs or A431-SCCs cells. We then analyzed the proteins secreted in their shared media, quantified the changes in proliferation, migration, invasion, angiogenesis and the gene expression of each cell type and compared each of the results to that of a mono-culture of the same cell type.

## Methods

All chemicals, if not noted separately, were purchased from Sigma-Aldrich, Munich, Germany.

### Donor specification

This study was conducted under the guidelines and with the approval of the ethical committees of the University of Heidelberg and of the medical association of the local district of Baden-Wuerttemberg, Germany. After the authors received informed consent, freshly excised subcutaneous adipose tissue of six women with an age-range of 24 to 48 years (median age 37.5 years) undergoing elective plastic surgery was used for isolation of ADSCs.

### ADSCs isolation and culture

#### Isolation of ADSCs

ADSCs were isolated from freshly excised subcutaneous adipose tissue or liposuction using a procedure modified from Hauner *et al*. [10]. In brief, the adipose tissue was washed in 1% bovine serum albumin (BSA)/phosphate-buffered saline (PBS), minced and digested enzymatically by collagenase (collagenase CLS; 220 U/mg, Biochrom AG, Berlin, Germany, 1.5 mg/ml, in 1% BSA/Krebs-Ringer-solution) for 45 minutes under constant shaking at 37°C. Mature adipocytes and connective tissue was separated by centrifugation (700 × g, seven minutes at room temperature). The sedimented cells were resuspended, passed through a 100 μm mesh filter (Neolab, Heidelberg, Germany) and washed twice with 1% BSA/PBS. After erythrocyte lysis (three minutes, 155 mM ammoniumchloride, 10 mM potassium bicarbonate, 0.1 mM EDTA) cells were washed again twice and plated at a density of 2 × 10^4^ cells/cm^2^ in an expansion medium (see below). After 24 hours the medium was changed to remove non-adhered cells.

### Expansion of ADSCs

ADSCs were cultivated in an expansion medium consisting of 60% Dulbecco’s modified Eagle’s medium (DMEM) low glucose (1 g/l D-glucose) (Invitrogen, Life Technologies, Darmstadt, Germany), 40% MCDB-201, 1 × ITS (insulin transferrin selenous acid) (Becton Dickinson, Heidelberg, Germany), 10^−8^ M dexamethasone, 0.1 mM ascorbic acid-2-phosphate, 2% fetal calf serum (FCS) (Biochrom), 100 U/ml penicillin (Biochrom), 0.1 mg/ml streptomycin (Biochrom), 10 ng/ml rhEGF and 10 ng/ml rhPDGF-BB (CellSystems, Troisdorf, Germany). The medium was changed every other day. Once the cells reached 70% confluence they were detached with 0.25% trypsin-EDTA (Biochrom) and replated with 3.5 × 10^3^ cells per cm^2^. ADSCs were incubated at 37°C with 5% CO_2_ and cultured to passage four.

### Determination of ADSCs stemness

#### Adipogenic differentiation and oil red staining

ADSCs were seeded in expansion medium at a density of 24,000 cells/cm^2^. After reaching 90% confluence, adipogenesis was induced by the alternated use of basal medium (10% FCS/DMEM) supplemented with IDI-mix (500 μM 3-isobutyl-1-methylxanthine; 1 μM dexamethasone; 1 μM indomethacin) for two days followed by basal medium plus 10 μg/ml insulin for one day. The induction cycle was repeated three times. To confirm the successful adipogenic differentiation, cytoplasmic triglyceride lipid droplets were stained with the Oil Red O staining method as described previously [11].

#### Osteogenic differentiation and alizarin red staining

After seeding with a density of 24,000 ADSC/cm^2^, cells were grown in expansion medium to 90% confluence. Osteogenic induction was initiated by changing the medium to DMEM containing 10% FCS, supplemented with 50 μM L-ascorbate-2-phosphate, 0.1 μM dexamethasone and 10 mM β-glycerophosphate disodium salt. On Day 42 calcium deposition was demonstrated histochemically by alizarin red staining as follows: monolayers of mineralized mesenchymal stem cells were washed twice with excess PBS and fixed with pre-chilled 70% ethanol for one hour at −20°C. After a short washing step with H_2_O, the cell layer was incubated with 40 mM alizarin red (pH 4.2) for one minute at room temperature. After aspiration of unincorporated dye, cells were washed twice with H_2_O and once with PBS before microscopic analysis.

#### Flow cytometry

ADSCs expanded to passage four were examined for surface marker expression using flow cytometry. The following monoclonal antibodies conjugated to fluorochromes were used: anti-CD13-APC, anti-CD29-PE, anti-CD31-FITC, anti-CD34-FITC, anti-CD44-APC, anti-CD45-FITC, anti-CD49a-PE, anti-CD63-FITC,-anti-CD73-PE, anti-CD90-APC, anti-CD105-FITC, anti-CD106-APC and anti-CD-166-PE (all from Becton Dickinson, Heidelberg, Germany). Isotype antibodies were included for all fluorochromes.

Cells were detached with 0.25% trypsin-EDTA, incubated with directly conjugated MAbs in FACS-buffer (1% FCS, 0.1% NaN_3_ in PBS) for 30 minutes on ice, washed twice with FACS buffer, and fixed with 1% paraformaldehyde/PBS. Cells were analyzed using a FACSCanto flow cytometry system (Becton Dickinson). Data acquisition was performed with Diva software (Becton Dickinson) and data were analyzed using FCS express V3 (De Novo Software).

### ADSC-SCC-co-culture

A431-SCCs were purchased from American Type Culture Collection (ATCC), Manassas, VA, USA, Catalog nr. CRL-1555. Primary SCCs from three different donors (two males, one female, median age 50 years) were purchased from Celprogen, San Pedro, CA, USA (Catalog nr. 36128–10). The primary SCCs were pooled initially, cultured for two passages and then applied to the co-culture system. Co-culture of tumor cells and ADSCs was performed in a transwell system. For that either 2 × 10^4^ SCCs or 2 × 10^4^ ADSCs were seeded onto a polyester membrane transwell-clear insert (Corning, pore size 0.4 μm) while the corresponding other cell type was seeded onto the bottom of a six-well cell culture plate in the same cell density. Cells were cultured up to five days in 4 ml expansion medium per well without medium change. Each day cell culture supernatants were harvested and the cell number was determined after trypzinization and trypan blue staining. SCCs as well as ADSCs alone - either in transwell inserts or on six-well culture plates - served as controls and were treated like the co-culture. For further analysis the exponential growth phase of the cells was determined and the supernatants of Day 4 were analyzed in a protein assay (human cytokine magnetic 30-Plex panel) while the corresponding cells were used for gene expression studies.

### Determination of cell proliferation

In order to obtain separate growth kinetics during the exponential growth phase for both, separately and co-cultured cells, cells of nine wells per condition (ADSCs alone, SCCs alone (A431- or primary SCCs), and both cell types in co-culture) were harvested with trypsin/EDTA once every 24 hours from Day 1 to Day 5. The cells were stained with trypan blue and the viable cells were counted with a Neubauer chamber. The generation time was calculated by the formula: G (hours) = (log2 × T)/(logY - logX) with T = time in culture (hours), Y = number of cells at the end of T, X = number of cells at the beginning of T. The results were evaluated using Student’s *t*-test.

### Analysis of cell migration

In order to determine the migration capacity of ADSC and SCCs alone (A431- or primary SCCs) and in co-culture, the QCM 24-Well Colorimetric Cell Migration Assay (Merck Millipore) was performed. For this purpose, cells of each cell type were seeded in expansion medium either on the bottom of the supplied 24-well plate (4,000 cells per well) or onto the membrane of the transwell insert (3,500 cells per insert). Cells were cultured separately for 24 hours before co-culture conditions (ADSCs on the well plate bottom, SCCs in the transwell inserts and vice versa) were established for a further 24 hours. Both cell types alone in the inserts served as controls. For evaluation of the assay, the medium was removed and the inserts transferred into new wells containing 400 μl cell stain for 20 minutes. The inserts were washed with water and the non-migrated cells were removed from the interior of the inserts with cotton-tipped swabs. The dried inserts were transferred into 200 μl of Extraction Buffer for 15 minutes and the optical density of 100 μl extracted dye was measured at 560 nm. The results were evaluated using Student’s *t*-test.

### *In vitro* analysis of invasive behavior

The invasion capacity of ADSC and SCCs was tested in a Cell Invasion Assay Kit (QCM ECMatrix Cell Invasion Assay, Merck Millipore). Cells of each cell type were seeded in expansion medium either on the bottom of the supplied 24-well plate (4,000 cells per well) or onto the membrane of the transwell insert (3,500 cells per insert). Cells were cultured separately for 24 hours (ADSCs) or 72 hours (SCCs) before co-culture - ADSCs on the bottom and SCCs in the inserts and vice versa - was induced for a further 72 hours. Both cell types alone in the inserts served as controls. Next, the medium was removed, the non-invading cells of the interior of the inserts were cleared with cotton-tipped swabs and the inserts transferred into 500 μl of staining solution for 20 minutes. Inserts were washed with water, air-dried and transferred into 200 μl of extraction buffer. The optical density of 150 μl extracted dye was measured at 560 nm. The results were evaluated using Student’s *t*-test.

### Quantitative real-time polymerase chain reaction (qrt-PCR)

The analysis of gene expression was carried out for 229 different genes in four main tumor associated areas: chemokines, apoptosis, molecular mechanisms of cancer and metastasis. Total RNA was isolated from ADSCs and SCCs, either cultured alone or in co-culture for four days, using the Trizol plus Kit (Life Technologies, Carlsbad, CA, USA). The RNA-concentration was calculated by Quant-iT RNA-Assay (Life Technologies) and 1 μg was subjected to cDNA synthesis by the High Capacity cDNA Reverse Transcription Kit (Life Technologies). Gene expression analysis was performed on a Step One Plus Instrument (Life Technologies) using TaqMan Real Time PCR technology. Gene expression was analyzed by using pre-designed TaqMan 96-well array plates each containing 92 different genes of interest and 4 endogenous controls with 10 ng cDNA per well (Human Molecular Mechanisms of cancer #4418806, Human Chemokines #4366072, Human Cellular Apoptosis Pathway #4418762, Human Tumor Metastasis #4418743, Life Technologies). In order to further investigate a potential epithelial to mesenchymal transition (EMT) of the cells during co-culture the gene expression of E- and N-cadherin was analyzed using specific TaqMan gene expression assays (Hs01023894 for E-cadherin, Hs00983056 for N-cadherin) with 10 ng of cDNA per sample. Calculating the difference between the cycle threshold (CT) of the genes of interest and the CT of the endogenous controls from the same sample provided delta-CT values.

### Human cytokine magnetic 30-plex panel

In order to quantify the level of 30 cytokines (CCL2, CCL3, CCL4, CCL5, CXCL-9, CXCL-10, EGF, Eotaxin, FGF-2, G-CSF, GM-CSF, HGF, IFN-α, IL-1β, IFN-γ, IL-1ra, IL-2, IL-2r, IL-4, IL-5, IL-6, IL-7, IL-8, IL-10, IL-12, IL-13, IL-15, IL-17, TNF-α and VEGF) and 8 different matrix metalloproteinases (MMP 1, 3, 7, 8, 9, 10, 12, 13) simultaneously in samples of ADSCs monoculture, SCCs monoculture, and ADSC-SCC-co-culture, a human cytokine magnetic 30-plex (LHC6003M and LHC6002, Life Technologies) and a human MMP magnetic Luminex Performance Assay were conducted according to the manufacturer’s instructions. Samples were analyzed with a Luminex 200 instrument (BioRad). The median fluorescent intensity was determined and the cytokine/MMP concentration ascertained based on the standard curves for each cytokine/MMP.

### Analysis of angiogenic properties

In order to determine the pro-angiogenic effect of ADSCs and A-431-SCCs or the primary SCCS alone or in co-culture, supernatants of each condition were collected at Day 4 of cell culture and analyzed for induction of tube formation in human umbilical vein endothelial cells (HUVEC) in an *in vitro* angiogenesis assay kit (Merck Millipore # ECM 625) according to the manufacturer’s instructions. In brief, wells of a 96-well plate were coated with an ECM Matrix solution, and 7,500 HUVEC cells were seeded onto the matrix in each well. The different conditioned media from ADSCs, A431-SCCs, pSCCs, or ADSC-SCC-co-cultures were added and incubated for 18 hours. Tube formation was visualized with a light microscope. A positive control was induced by Phorbol 12-myristate 13-acetate (PMA) (Abcam, Cambridge, UK; no. ab120297).

## Results

### Determination of stemness

The stemness of the applied ADSCs was determined according to the minimal consensus criteria for mesenchymal stem cells [12,13] by analysis of distinct surface markers in flow cytometry and analysis of adipogenic and osteogenic differentiation with Oil Red and alizarin red staining, respectively.

#### Flow cytometry

**Figure 1:**
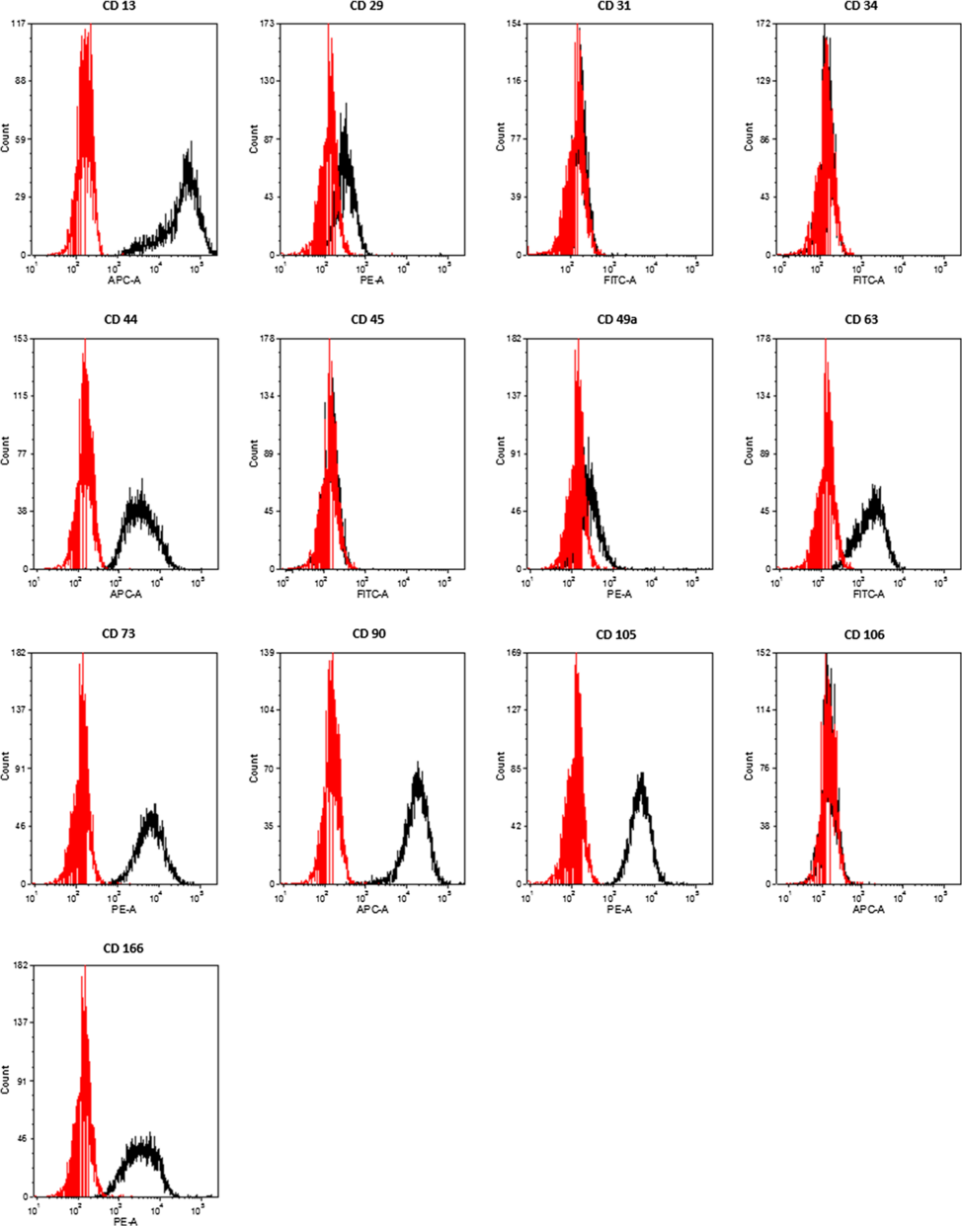
**Flow cytometry of pooled ADSCs from donors 1 to 6.** Red lines show isotype controls, black lines show pooled ADSCs. ADSCs were positive for CD13, CD29, CD44, CD49a, CD63, CD73, CD90, CD105 and CD166. ADSCs were negative for CD31, CD34, CD45 and CD106. ADSCs, adipose tissue derived stem cells.

ADSCs were positive for CD13, CD29, CD44, CD49a, CD63, CD73, CD90, CD105 and CD166. ADSCs were negative for CD31, CD34, CD45 and CD106 (Figure 1).

#### Differentiation

**Figure 2:**
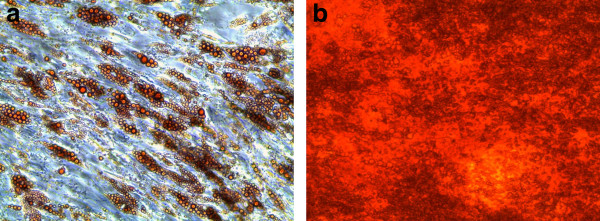
**Representative light microscopical pictures of adipogenically and osteogenically differentiated ADSCs.** Magnification 10x. **(a)** Intracellular lipid droplets stained by oil red method as a marker of adipogenic differentiation on Day 14 of differentiation. **(b)** Extracellular calcium deposition stained with alizarin red as a marker for osteogenic differentiation on Day 42 of differentiation. Undifferentiated controls are not shown. ADSCs, adipose tissue derived stem cells.

Adipogenic and osteogenic differentiations were induced to evaluate the multipotent differentiation potential. In all donors adipogenically induced cells showed a significantly higher oil red staining than non-induced control cells (Figure 2a). Osteogenically differentiated ADSC showed significantly higher extracellular calcium deposition than non-induced control cells, analyzed with alizarin red stain (Figure 2b). The cells, therefore, meet the minimal consensus criteria for mesenchymal stem cells [12,13].

### Proliferation

**Figure 3:**
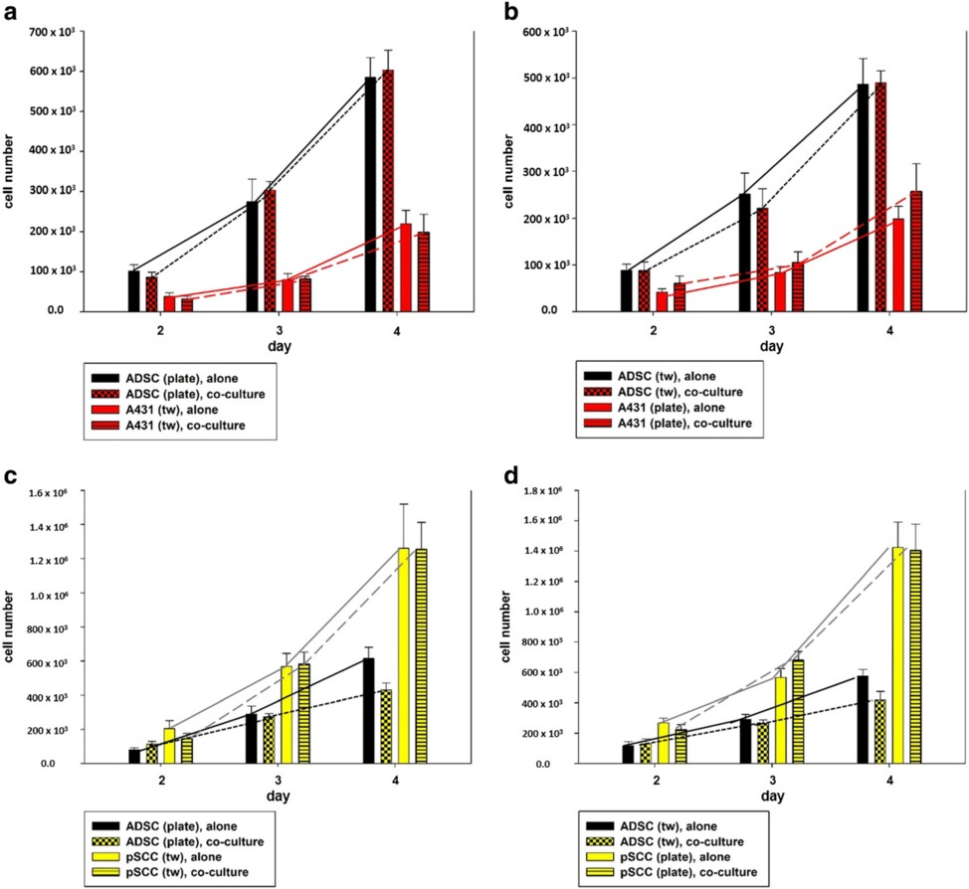
**Effect of ADSC-SCC-co-culture on cells’ proliferative activity. (a)** The growth of ADSCs on the plate significantly increased (*P* = 0.038) in co-culture with A431-SCCs in the transwell insert compared to mono-culture. The growth of co-cultured A431-SCCs in the transwell insert was not significantly affected (*P* >0.05). **(b)** ADSC-SCC-co-culture with A431-SCCs on the six-well plate and ADSCs in the transwell insert did not significantly affect cell growth of both cell types (*P* >0.05). **(c)** ADSC-SCC-co-culture with ADSCs on the six-well plate and pSCCs in the transwell insert significantly reduced ADSCs’ growth compared to mono-culture (*P* = 0.03). The proliferative activity of pSCCs in co-culture was not affected (*P* >0.05). **(d)** ADSC-SCC-co-culture with pSCCs on the six-well plate and ADSCs in the transwell insert significantly decreased the growth of pSCCs (*P* <0.001), that of co-cultured ADSCs was not significantly affected (*P* >0.05). ADSC, adipose tissue derived stem cells; SCCs, squamous cell carcinoma cells.

Cell proliferation was determined by analyzing the cells’ doubling time during the exponential growth phase (in general from days 2 to 4) (Figure 3). Data are given as means with standard deviation (SD). Changes in cell numbers per time are given in Figure 3.

#### ADSCs-A431-SCCs-co-culture

When ADSCs were cultured alone on a regular culture surface in the six-well plate, the mean doubling time was 20 hours (SD 11), when cultured alone in a transwell insert, the mean cell doubling time was 17 hours (SD 5). A431-SCCs cultured alone in a six-well plate showed a doubling of cell number after a mean of 17 hours (SD 7), in a transwell insert after 16 hours (SD 4). When co-cultured in a transwell system, the growth of ADSCs on the six-well plate significantly increased to a mean doubling time of 14 hours (SD 3, *P* = 0.038) (Figure 3a). In the transwell insert the growth rate of co-cultured ADSCs slowed down to a mean doubling time of 19 hours (SD 9), that of A431-SCCs slowed down to a mean doubling time of 25 hours (SD 13) in the six-well plate, and to 21 hours (SD 8) in the transwell insert (Figure 3b). These changes, however, were not statistically significant (*P* >0.05).

#### ADSCs-pSCCs-co-culture

When ADSCs were cultured alone on a regular culture surface in the six-well plate the mean doubling time was 26 hours (SD 8), when cultured alone in a transwell insert, the mean cell doubling time was 28 hours (SD 7). Primary SCCs cultured alone in a six-well plate showed a doubling of cell number after a mean of 18 hours (SD 3), in a transwell insert after 21 hours (SD 6).

Co-culturing ADSCs with primary SCCs resulted in a decreased proliferation determined by a significantly longer doubling time in ADSCs cultured on the six-well plate (42 hours, SD 13, *P* = 0.03) or in the transwell system (51 hours, SD 25) (Figure 3c). In co-culture, primary SCCs also significantly decreased their proliferation (25 hours, SD 4, *P* <0.001), if cultured on the plate (Figure 3d). If ADSCs were cultured in the transwell insert, there was no significant change in proliferative activity (22 hours (SD 4), *P* >0.05).

### Quantitative real time-PCR

Co-cultured ADSCs and SCCs (A431-SCCs and primary SCCs) showed strong differences in the gene expression levels compared to a mono-culture of ADSCs or SCCs. Results were greatly similar for the two analyzed co-culture systems (ADSCs on plate/SCCs in transwell insert, and ADSCs in transwell insert/SCCs on plate). To facilitate clear data presentation, only results of the ADSCs on plate/SCCs in transwell insert - co-culture, and (except for E- and N-Cadherin) changes in gene expression 2.5-fold or greater are displayed.

#### Co-culture of A431-SCCs and ADSCs

In the ADSCs, a strong increase of the gene expression level could be found in CSF-3 (258-fold), CXCL6 (129-fold), IL-1β (119-fold), MMP-3 (108-fold), IL-6 (88-fold), PTGS2 (74-fold), CXCL1 (54-fold), CCL8 (42-fold), CSF-2 (38-fold), CCL20 (32-fold), IL-8 (29-fold), CXCL3 (27-fold), CXCL2 (16-fold), CXCL12 and IL-1α (12-fold), CCL7 (11-fold), CCL2 and CXCL5 (9.4-fold), CCL13 (8-fold), LYPD3 and WISP-1 (6.1-fold) and FGFR4 (5.4-fold).

To a lesser extent the expression of NR4A3 (4.7-fold), TLR-2 (4.6-fold), CXCL11, MCAM, STAT4 and NFKBIA (4.0-fold), MMP-2 and TYMP (3.4-fold), PIK3CD and PPBP (3.3-fold), NFKB2 and RELB (2.7-fold), FGF2 (2.5-fold), GPR81 (2.4 fold), TNF and CCRL1 (2.3-fold) was also up-regulated (see Table 1, part 1 and Additional file 1: Table S1, A, part 1).

**Table 1:** Changes in the gene expression levels of ADSCs and A431-SCCs in co-culture compared to mono-culture

**Gene**	**Fold ADSC mono-/co-culture**	**Up-/down--regulation**	**Main impact of gene product**
*Part 1. Changes in the gene expression of ADSCs*			
*E-Cadherin*	n.d.	→	EMT, i, m, ms
*N-Cadherin*	1.0 (0.0)	→	EMT
*CCL2*	9.4 (5.0)	↑	a, i, m, ms, p, EMT
*CCL7*	11 (3.8)	↑	a, i, m, ms, p
*CCL8*	42 (15)	↑	a, i, m, p
*CCL13*	8.0 (0.0)	↑	a, i, m, p
*CCL20*	32 (23)	↑	im
*CSF-2*	38 (20)	↑	a, i, m
*CSF-3*	258 (2.3)	↑	im
*CXCL1*	54 (16)	↑	a, i, m, ms, p
*CXCL2*	16 (0.0)	↑	a, i, m
*CXCL3*	27 (7.8)	↑	a, i, m
*CXCL5*	9.4 (5.0)	↑	a, i, m
*CXCL6*	129 (0.2)	↑	a, i, m, ms, p
*CXCL11*	4.0 (0.0)	↑	a, i, m
*CXCL12*	12 (5.7)	↑	a, i, m
*IL-1α*	12 (5,6)	↑	a, ms
*IL-1β*	119 (100)	↑	a, ms
*IL-6*	88 (84)	↑	p
*IL-8*	29 (25)	↑	a
*LYPD3*	6.1 (2.9)	↑	i, ms
*MMP-3*	108 (32)	↑	m, i, ms
*NR4A3*	4.7 (2.8)	↑	p
*PTGS2*	74 (39)	↑	a, i, im, m, ms, p
*TLR-2*	4.6 (2.5)	↑	a, m, i
*WISP-1*	6.1 (2.9)	↑	m, im
**Gene**	**Fold A431-SCC mono-/co-culture**	**Up-/down-regulation**	**Main impact of gene or gene product**
*Part 2. Changes in the gene expression of A431-SCCs*			
*E-Cadherin*	*	↓↓	EMT, i, m, ms
*N-Cadherin*	98 (2.3)	↑	EMT
*CXCL9*	4.4 (0.2)	↓	a
*CXCL10*	4.8 (0.1)	↓	a
*CTKS*	4.1 (2.9)	↑	m, i, ms
*FN1*	2.5 (1.3)	↑	m, i
*HGF*	20 (12)	↑	a, m, i,
*ITGB3*	10 (5.1)	↑	m, i, ms
*MMP-1*	9.4 (5.1)	↑	m, i, ms
*MMP-2*	3.1 (1.5)	↑	m, i, ms
*MMP-3*	191 (89)	↑	m, i, ms
*MMP-7*	4.8 (2.4)	↑	m, i, ms
*MMP-9*	3.4 (0.2)	↓	m, i, ms
*PTGS2*	2.7 (0.8)	↑	a, i, im, m, ms, p
*TWIST-1*	11 (3.8 )	↑	ms

*In co-culture with ADSCs *E-Cadherin* expression is down-regulated in primary SCC to undetectable levels.

*GUSB* was used as referring housekeeping-gene. Except for *E*- and *N-Cadherin* only changes of 2.5-fold or higher are displayed. Part 1 displays the changes in the gene expression levels of ADSCs. Part 2 shows the changes in the gene expression levels of A431-SCCs. Arrows mark an up- (↑) or down-regulation (↓) of the gene expression compared to the referring mono-culture. Abbreviations of main impact: a, angiogenesis; i, invasion, im, impaired anti-tumor response; m, migration; ms, metastasis; p, proliferation.;Furthermore a mild down-regulation of E2F1 (3.2-fold), CCND1, CCND3, CYCS and DVL1 (2.7-fold) and SPP1 (2.6-fold) could be determined.

When A431-SCCs were co-cultured with ADSCs the following genes were strongly up-regulated in the A431-SCCs: *MMP-3* (191-fold), *N-Cadherin* (97-fold), *HGF* (20-fold), *TWIST1* (11-fold), *ITGB3* (10-fold), *MMP-1* (9.4-fold), and others to a lesser extent: *MMP-7* (4.8-fold), *CTSK* (4.1-fold), *MMP-2* (3.1-fold), *CYCS* (3-fold), *PTGS2* (2.7-fold), *PPBP*, *IKBKE* and *ETV4* (2.5-fold), *CCL20* and *FN1* (2.4-fold) and *MCAM* (2.3-fold). In addition, *N-Cadherin*-expression in A431-SCCs is strongly up-regulated in co-culture (71-fold) while there is no significant change in co-cultured ADSCs (1.1-fold).

ADSCs did not express *E-Cadherin* in mono-culture; in co-culture however, *E-Cadherin* expression was strongly up-regulated. In co-cultured A431-SCCs, *E-Cadherin* was slightly down-regulated (1.2-fold). Furthermore, the expression of *CXCL9* (4.4-fold), *CXCL10* (4.8.-fold), *FOS* (4-fold), *MMP-9* (3.4-fold) and *PMAIP1* (3.1-fold) was also down-regulated (see Table 1, part 2 and Additional file 1: Table S1, A, part 2).

#### Co-culture of primary SCCs and ADSCs

In the ADSCs a strong increase of the gene expression level could be found in IL-6 (65-fold), CXCL6 (64-fold), CSF-3 (63-fold), IL-8 and CCL20 (32-fold), CCL28 (27-fold), PTGS2 (22-fold), TLR-2 (21-fold), WISP-1 (20-fold), CCL5, CXCL1, CXCL3 and IL-1β (16-fold), MMP-3, IL-1α and CSF-2 (13-fold), CXCL5 (8.1-fold), VEGFA (6.7), KISS1R (5.6-fold), CXCL14, ITGA2B and SERPINE (5.4-fold). To a lesser extent the expression of CCL2 and CXCL2 (4-fold), CCL7, CCL8 and PIK3CD (3.4-fold), CXCR4 (3.1-fold), CXCL11, CXCL12, CCL3, CCL13, FGFR4, MMP-2, MMP-9 and TPBG (2.7-fold) was also up-regulated (see Table 2, part 1 and Additional file 1: Table S1, B, part 1). ADSCs did not express E-Cadherin in mono-culture, and it could not be determined in co-cultured ADSCs either. There was no significant change of N-Cadherin-expression in ADSCs in co-culture (1.1-fold). A mild down-regulation of FOS and BCL2 (3-fold) could be determined.

**Table 2:** Changes in the gene expression levels of ADSCs and pSCCs in co-culture compared to mono-culture

**Gene**	**Fold ADSC mono-/co-culture**	**Up-/down-regulation**	**Main impact of gene or gene product**
*Part 1. Changes in the gene expression of ADSCs*			
*E-Cadherin*	n.d.	n.a.	EMT, i, m, ms
*N-Cadherin*	0.9 (0.0)	→	EMT
*CCL5*	16 (0.1)	↑	a, i, ms, p
*CCL20*	32 (0.4)	↑	im
*CCL28*	27 (7.6)	↑	a, i, ms, p
*CSF-2*	13 (4,3)	↑	a, i, m
*CSF-3*	63 (0.2)	↑	im
*CXCL1*	16 (0.0)	↑	a, i, m, ms, p
*CXCL2*	4.0 (0.0)	↑	a, i, m
*CXCL3*	16 (0.1)	↑	a, i, m
*CXCL5*	8.1 (0.0)	↑	a, i, m
*CXCL6*	64 (0.2)	↑	a, i, m, ms, p
*CXCL14*	5.4 (1.9)	↑	a
*IL-1α*	13 (3.7)	↑	a, ms
*IL-1β*	16 (0.1)	↑	a, ms
*IL-6*	65 (0.8)	↑	p
*IL-8*	32 (0.7)	↑	a
*ITGA2B*	5.4 (1.9)	↑	m, i, ms
*KISS1R*	5.6 (2.0)	↑	ms
*MMP-3*	13 (3.8)	↑	m, i, ms
*PTGS2*	22 (7.8)	↑	a, i, im, m, ms, p
*SERPINE1*	5.4 (2.0)	↑	i, a, m
*TLR-2*	21 (7.6)	↑	a, ms
*VEGFA*	6.7 (1.9)	↑	a
*WISP-1*	20 (12)	↑	m, im
**Gene**	**Fold primary SCC mono-/co-culture**	**Up-/down-regulation**	**Main impact of gene or gene product**
*Part 2. Changes in the gene expression of primary SCCs*			
*E-Cadherin*	1.5 (0.1)	→	EMT, i, m, ms
*N-Cadherin*	3.0 (0.0)	↓	EMT
*CCND2*	4.7 (3.9)	↑	i

GUSB was used as the referring housekeeping-gene. Except for E- and N-Cadherin only changes of 2.5-fold or higher are displayed. Part 1 displays the changes in the gene expression levels of ADSCs. Part 2 shows the changes in the gene expression levels of pSCCs. Arrows mark an up- (↑) or down-regulation (↓) of the gene expression compared to the referring mono-culture. Abbreviations of main impact: a, angiogenesis; i, invasion; im, impaired anti-tumor response; m, migration; ms, metastasis; p, proliferation.

When primary SCCs were co-cultured with ADSCs the *CCND2* gene was up-regulated (4.7-fold) and the following genes were down-regulated: *TNF* (2.7-fold) and *CCL5* (3.0-fold) (see Table 2, part 2 and Additional file 1: Table S1, B, part 2). *E-Cadherin*-expression and *N-Cadherin* gene expression was not significantly changed during co-culture with ADSCs (1.5-fold, and 3.0-fold).

### Multiplex protein analysis

#### Co-culture of A431-SCCs and ADSCs

When ADSC were co-cultured with A431-SCCs cells (see Table 3), a very strong increase in the protein concentration in the conditioned medium could be detected for G-CSF (357-fold), INF-α (118-fold), GM-CSF (106-fold), MMP-9 (45-fold), IL-6 (43-fold), MMP-3 (6.8-fold), and CCL2 (8-fold). VEGF (6.0-fold), MMP-10 (3.4-fold), INF-γ (3.9-fold), CCL4 (3.5-fold), IL-7 (4.9-fold), IL-8 (5.4-fold), IL-13 (3.5-fold) and IL-12 (2.8-fold) were only moderately increased. The concentrations of bFGF, CCL3, CXCL10, Eotaxin, HGF, IL-4, IL-10, IL-15, IL-17, MMP-1 and TNF-α were not significantly changed compared to the mono-culture of ADSCs (<2.5-fold change).

**Table 3:** Changes in the protein expression levels of ADSCs and A431-SCCs in co-culture compared to mono-culture

**Protein**	**Pg/ml, ADSC mono-culture**	**Pg/ml, A431 mono-culture**	**Pg/ml, co-culture**	**Fold ADSC mono-/co-culture**	**Fold A431 mono-/co-culture**
bFGF	4.7 (1.8)	7.8 (1.6)	7.5 (2.0)	1.6 - →	1.0 - →
CCL2	2,498 (821)	168 (23)	21,096 (4,041)	8.5 - ↑	126 - ↑
CCL3	24.1 (1.4)	22.4 (1.9)	39.2 (2.9)	1.6 - →	1.7 - →
CCL4	5.8 (3.6)	2.7 (2.5)	20 (2.7)	3.5 - ↑	7.4 - ↑
CCL5	n.d.	13 (2.5)	n.d.	- - →	- -↓
CXCL9	n.d.	n.d.	411 (24)	- - ↑	- - ↑
CXCL10	4.9 (0.3)	4.6 (0.1)	6.4 (0.3)	1.3 - →	1.2 - →
Eotaxin	1.7 (0.1)	1.6 (0.3)	1.8 (0.6)	1.1 - →	1.2 - →
G-CSF	98 (19)	1,225 (135)	34,941 (4,830)	357 - ↑	29 - ↑
GM-CSF	1.3 (0.2)	100 (8.9)	133 (28)	106 - ↑	1.3 - →
HGF	6,892 (1,788)	34 (15)	4,049 (746)	0.6 - →	118 - ↑
IL-1b	n.d.	6.1 (3.2)	11 (4.9)	- - ↑	1.8 - →
IL-2	5.6 (1.4)	5.9 (0.8)	6.2 (0.9)	- - →	- - →
IL-4	22 (1.3)	19 (0.7)	31 (1.8)	1.4 - →	1.6 - →
IL-6	313 (80)	45 (4.1)	13,563 (917)	43 - ↑	301 - ↑
IL-7	20 (7.5)	33 (8.3)	100 (19)	4.9 - ↑	3.0 - ↑
IL-8	9,213 (3,483)	3,898 (404)	49,441 (1,572)	5.4 - ↑	13 - ↑
IL-10	7.3 (0.3)	7.8 (0.3)	16 (0.6)	2.2 - →	2.0 - →
IL-12	27.4 (8.0)	35.2 (7.8)	76 (5.1)	2.8 - ↑	2.2 - →
IL-13	18 (1.0)	20 (1.3)	27 (1.1)	3.5 - ↑	1.4 - →
IL-15	36 (11)	38 (19)	33 (20)	0.9 - →	0.9 - →
IL-17	11.2 (0.7)	9.6 (0.7)	14 (1.2)	1.3 - →	1.5 - →
IL-1Ra	n.d.	1,084 (82)	687 (65)	0.6 - →	0.6 - →
IL-2R	n.d.	109 (0.0)	50 (31)	- - ↑	0.5 - →
INF-α	84 (16)	40 (5.4)	268 (14)	118 - ↑	6.7 - ↑
INF-γ	2.3 (0.9)	5.7 (1.4)	8.8 (2.4)	3.9 - ↑	1.5 - →
MMP-1	33,056 (5,448)	302 (33)	43,274 (15,154)	1.3 - →	143 - ↑
MMP-3	16,089 (3,424)	72 (7)	109,950 (47,520)	6.8 - ↑	1,527 - ↑
MMP-7	n.d.	n.d.	131 (42)	- - ↑	- - ↑
MMP-9	156 (18)	533 (57)	6,987 (15,036)	45 - ↑	13 - ↑
MMP-10	357 (67)	70 (2.3)	1,202 (333)	3.4 - ↑	17 - ↑
TNF-α	2.7 (1.3)	2.5 (1.0)	4.1 (2.0)	1.5 - →	1.7 - →
VEGF	61 (11)	132 (22)	362 (40)	6.0 - ↑	2.8 - ↑

CXCL9 and MMP-7 could only be detected in co-culture. Standard deviation is given in brackets. Results from 0 to 9.9 are shown with one decimal, results 10 or higher are displayed without decimals. n.d., not detectable; − −, not applicable.

Exclusively in co-culture ADSCs were exposed to CXCL9 (441 pg/ml, SD 24), IL-1β (11 pg/ml, SD 4.9), IL-1Ra (687 pg/ml (SD 65)), IL-2R (50 pg/ml, SD 31) and MMP-7 (131 pg/ml, SD 42).

With regard to A431-SCCs mono-culture, the co-culture with ADSCs led to a very strong increase in MMP-3 (1527-fold), MMP-1 (143-fold), IL-6 (301-fold), CCL2 (126-fold), HGF (118-fold), G-CSF (29-fold), MMP-10 (17-fold), MMP-9 (13-fold), and IL-8 (13-fold), and a moderate increase of CCL4 (7.4-fold), INF-α (6.7-fold), IL-7 (3.0-fold) and VEGF (2.8-fold). Compared to a A431-SCC-mono-culture, co-culturing with ADSCs did not significantly change the concentration of bFGF, CCL3, CXCL10, Eotaxin, GM-CSF, IL-1β, IL-4, IL-10, IL-12, IL-13, IL-15, IL-17, IL-1Ra, IL-2R and TNF-α. In contrast to that, CCL5 was down-regulated to a no longer detectable level in co-cultured A431-SCCs.

CXCL-9 (411 pg/ml, SD 24) and MMP-7 (131 pg/ml, SD 42) were exclusively produced in co-culture but neither in the mono-culture of A431-SCCs nor of ADSCs.

#### Co-culture of primary SCCs and ADSCs

When ADSCs were co-cultured with primary SCCs cells (see Table 4), a strong increase in the protein concentration in the conditioned medium could be detected for G-CSF (77-fold) and GM-CSF (10-fold), and a moderate increase for IL-6 (5.5-fold), CCL4 (3.8-fold), IL-7 (3.4-fold), IL-8 (3.0-fold) and IL-2 (2.5-fold). Exclusively in co-culture, ADSCs were exposed to CCL5 and CXCL9.

**Table 4:** Changes in the protein expression levels of ADSCs and pSCCs in co-culture compared to mono-culture

**Protein**	**Pg/ml, ADSC mono-culture**	**Pg/ml, pSCC mono-culture**	**Pg/ml, co-culture**	**Fold ADSC mono-/co-culture**	**Fold pSCC mono-/co-culture**
bFGF	3.9 (1.9)	5.3 (1.1)	6.6 (1.8)	1.7 - →	1.3 - ↑
CCL2	2,768 (109)	565 (83)	5,707	2.1 - →	10 - ↑
CCL3	26 (1.7)	25 (1.5)	32 (4.6)	1.2 - →	1.3 - →
CCL4	2.9 (2.0)	2.0 (0.0)	11 (4.9)	3.8 - ↑	5.4 - ↑
CCL5	n.d.	216 (32)	95 (8.4)	- - ↑	0.4 - →
CXCL9	n.d.	n.d.	208 (14)	- - ↑	- - ↑
CXCL10	4.7 (0.2)	5.7 (0.5)	7.0 (0.8)	1.5 - →	1.2 - →
Eotaxin	1.7 (0.4)	1.8 (0.3)	2.1 (0.2)	1.3 - →	1.2 - →
GM-CSF	1.0 (0.1)	13 (0.9)	32 (4.6)	10 - ↑	0.8 - →
G-CSF	40 (23)	n.d.	3,076 (330)	77 - ↑	- - ↑
HGF	6,125 (317)	72 (31)	3,661 (174)	0.6 - →	51 - ↑
IL-1b	n.d.	n.d.	6.2 (1.2)	- - ↑	- - ↑
IL-2	4.7 (0.7)	5.4 (0.6)	5.2 (0.9)	2.5 - ↑	1.0 - →
IL-4	22 (0.9)	21 (0.6)	29 (2.1)	1.3 - →	1.4 - →
IL-6	349 (40)	4.0 (1.4)	1,912 (396)	5.5 - ↑	480 - ↑
IL-7	16 (1.2)	n.d.	53 (23)	3.4 - ↑	- - ↑
IL-8	11,219 (652)	8,825 (791)	34,032 (1,805)	3.0 - ↑	3.9 - ↑
IL-10	7.3 (0.0)	6.4 (0.6)	9.5 (0.7)	1.3 - →	1.5 - →
IL-12	28 (5.1)	29 (6.0)	54 (11)	1.9 - →	1.9 - →
IL-13	18 (1.6)	15 (0.8)	20 (3.0)	1.1 - →	1.4 - →
IL-17	11 (1.1)	10 (0.6)	14 (1.4)	1.3 - →	1.4 - →
INF-α	102 (5.6)	61 (6.6)	156 (12)	1.5 - →	2.6 - ↑
INF-γ	2.2 (0.5)	3.0 (1.4)	6.1 (3.5)	1.6 - →	1.0 - →
MMP-1	44,830 (2,372)	n.d.	24,473 (1,564)	1.8 - →	- - ↑
MMP-3	2,288 (268)	47 (2.6)	4,354 (386)	1.9 - →	92 - ↑
MMP-9	110 (5.2)	275 (19)	174 (11)	1.6 - →	1.6 - →
MMP-10	149 (14)	17 (3.5)	248 (13)	1.7 - →	14 - ↑
TNF-α	2.1 (0.5)	3.2 (0.9)	3.2 (1.9)	1.6 - →	1.0 - →
VEGF	63 (8.7)	26 (8.1)	117 (30)	1.9 - →	4.6 - ↑

CXCL9 and IL-1β could only be detected in co-culture. Standard deviation is given in brackets. Results from 0 to 9.9 are shown with one decimal, results 10 or higher are displayed without decimals. n.d., not detectable, − −, not applicable.

For ADSCs, no major changes in the protein level of bFGF, CCL2, CCL3, CXCL10, Eotaxin, MMP-1, MMP-3, MMP-9, MMP-10, HGF, INF-α, INF-γ, IL-4, IL-10, IL-12, IL-13, IL-17, TNF-α and VEGF (<2.5-fold change) could be detected in co-culture compared to mono-culture.

With regard to SCCs mono-culture, the co-culture with ADSCs led to a very strong increase in IL-6 (480-fold), MMP-3 (92-fold), HGF (51-fold), MMP10 (14-fold), CCL2 (10-fold), and a moderate increase in CCL4 (5.4-fold), VEGF (4.6-fold), IL-8 (3.9-fold) and INF-α (2.6).

Compared to a pSCC-mono-culture, co-culturing with ADSCs did not significantly change the concentration of bFGF, CCL3, CCL5, CXCL10, Eotaxin, GM-CSF, IL-2, IL-4, IL-10, IL-12, IL-13, IL-17, IL-1Ra, IL-2R, INF-γ, MMP-9 and TNF-α (<2.5-fold change).

Exclusively in co-culture, primary SCCs were exposed to IL-7 (53 pg/ml, SD 23), G-CSF (3,076, SD 330) and MMP-1 (24,473 pg/ml, SD 1,564).

CXCL-9 (208 pg/ml, SD 14) and IL-1β (6.2 pg/ml, SD 1.2) were exclusively produced in co-culture of ADSCs and primary SCCs but not in mono-culture of both cell types.

### Migration

**Figure 4:**
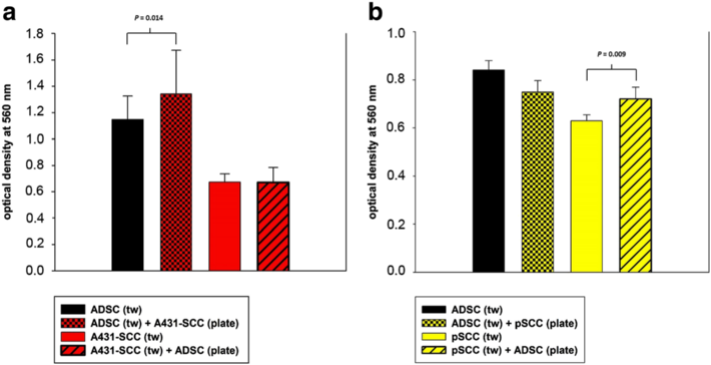
**Migration assay. a)** Migratory capacity of the ADSCs (black bar) and A431-SCCs (red bar) alone and in co-culture, measured as the level of optical density at 560 nm, with a standard deviation (SD). When co-cultured with A431-SCCs (black bar with red checkerboard pattern) ADSCs showed a significantly higher migration as in mono-culture (*P* = 0.014). Co-culture of A431-SCCs with ADSCs (red bars with black diagonal slashes) does not lead to a significant change in the migratory properties of A431-SCCs (*P* >0.05). **b)** Migratory capacity of the ADSCs (black bar) and pSCCs (yellow bar) alone and in co-culture, measured as the level of optical density at 560 nm, with a SD. When co-cultured with pSCCs (black bar with yellow checkerboard pattern) ADSCs showed a higher migration as in mono-culture (*P* >0.05). Co-culture of pSCCs with ADSCs (yellow bars with black diagonal slashes) resulted in a significantly increased migration of pSCCs (*P* = 0.009). ADSC, adipose tissue derived stem cells; pSCCs, primary squamous cell carcinoma cells; SCCs, squamous cell carcinoma cells.

The migration through the transwell pores could already be detected when ADSC, A431-SCCs, or primary SCCs were cultured alone; however, when co-cultured with A431-SCCs the migration of ADSC was significantly increased about 17% (*P* = 0.012), while that of the A431-SCCs was not changed compared to the mono-culture (*P* >0.05) (Figure 4a). In co-culture with ADSCs the migratory capacity of the primary SCCs was also significantly increased by about 15% (*P* = 0.009) while that of the ADSCs remained unchanged (Figure 4b).

### Invasion

**Figure 5:**
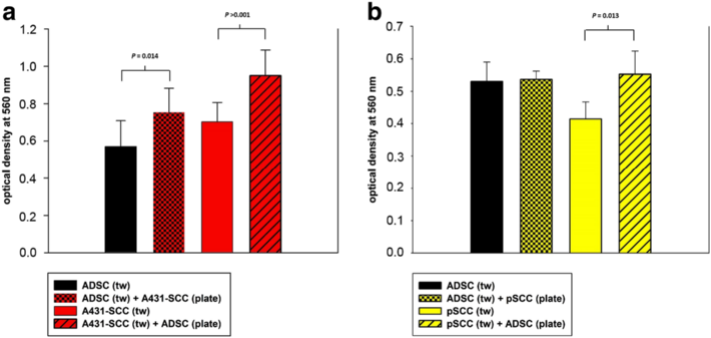
**Invasion assay. a)** Invasive behavior of ADSCs (black bar) and A431-SCCs (red bar) alone and in co-culture, measured as the level of optical density at 560 nm with a standard deviation (SD). When co-cultured with A431-SCCs (black bar with red checkerboard pattern) ADSCs showed a significantly higher invasive capacity as in mono-culture (*P* = 0.014). Co-culture of SCCs with ADSCs (red bars with black diagonal slashes) also leads to a significant increase in the invasive behavior of A431-SCCs (*P* <0.001). **b)** Invasive behavior of ADSCs (black bar) and pSCCs (yellow bar) alone and in co-culture, measured as the level of optical density at 560 nm, with a SD. When co-cultured with pSCCs (black bar with yellow checkerboard pattern) ADSCs did not significantly change their invasive capacity compared to mono-culture. Co-culture of pSCCs with ADSCs (yellow bars with black diagonal slashes), however, leads to a significant increase in the invasive behavior of pSCCs (*P* = 0.013). ADSC, adipose tissue derived stem cells; pSCCs, primary squamous cell carcinoma cells; SCCs, squamous cell carcinoma cells.

ADSCs and A431-SCCs showed invasive behavior by actively digesting the extracellular matrix blocking the transwell pores and migrating to the lower surface of the transwell inserts’ floor. This was significantly increased by a mean of 33% in the co-cultures for ADSCs (*P* = 0.014) and significantly increased by a mean of 35% for SCCs (*P* <0.001), respectively (Figure 5a). When co-culturing primary SCCs with ADSCs the invasive behavior of the primary SCCs was significantly increased by a mean of 33% compared to the culture of primary SCCs alone (*P* = 0.038). The invasive behavior of ADSCs in this context was not changed by co-culturing with primary SCCs (Figure 5b).

### Angiogenesis

**Figure 6:**
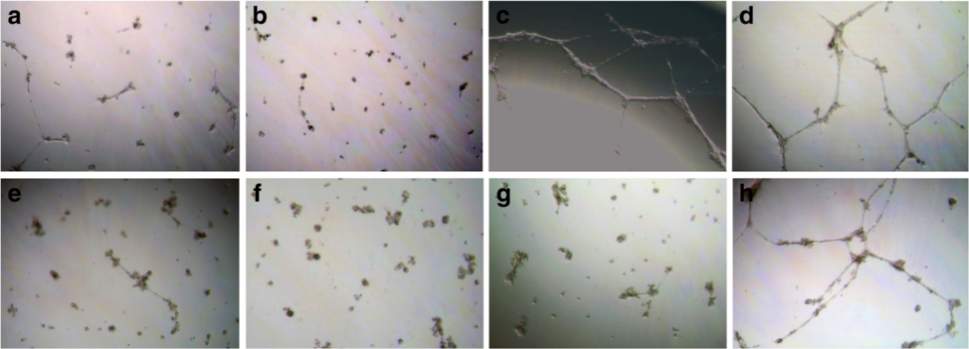
**Induction of angiogenesis.** Angiogenesis assay with incubation of HUVEC cells with conditioned media from mono-cultured ADSCs **(a, e)**, A431-SCCs **(b)**, pSCCs **(f)**, co-cultured ADSC-A431-SCCs **(c)**, and co-cultured ADSCs-pSCCs **(g)**. Controls **(d, h)** were induced with PMA. Tube formation could be detected with conditioned media from co-cultured ADSC-A431-SCCs, slightly from co-cultured ADSCs-pSCCs, and mono-cultured ADSCs or A431-SCCs. No significant angiogenesis could be detected when conditioned media from pSCC- or A431-SCC-mono-culture was added to the system. ADSC, adipose tissue derived stem cells; HUVEC, human umbilical vein endothelial cells; PMA, phorbol 12-myristate 13-acetate; pSCCs, primary squamous cell carcinoma cells; SCCs, squamous cell carcinoma cells.

Tube formation could be detected after incubation of HUVEC cells with conditioned media from co-cultured ADSC-A431-SCCs and slightly from co-cultured ADSCs-pSCCs, and mono-cultured ADSCs or A431-SCCs. No significant angiogenesis could be detected when conditioned media from pSCC- or A431-SCC-mono-culture was added to the system (Figure 6).

## Discussion

ADSCs are a promising future tool in skin regenerative medicine, providing many so far suggested positive effects, such as increasing the dermal collagen content, supporting neoangiogenesis or inhibiting melanin synthesis and the activity of skin matrix degrading enzymes [1,3-5,14]. These effects are mainly due to the paracrine activity of the transplanted stem cells, reflecting the reaction of the surrounding cells, such as dermal fibroblasts or melanocytes, towards secreted cytokines and growth factors. *In vivo*, however, there is always a risk of malignant cells being present in the vicinity of the transplanted ADSCs. These malignant cells would also be in direct contact with the released proteins, and thus, prone to be influenced by the ADSCs secretome. Nevertheless, research about the possible interactions of ADSCs and skin tumor cells is still not in the focus of most skin regeneration projects. The present study is the first to show that the co-culture of human SCCs and human ADSCs leads to a significant change in the gene expression profile of both cell types, as well as a remarkable change in the secreted protein levels. Additionally, ADSCs increased their migration towards SCCs, and invasiveness rose in both cell types, with the latter being an important indicator for a possible rise of local destruction and occurrence of metastases. Furthermore, some proteins, such as MMP-7, IL-1β or CXCL9, were exclusively produced in ADSC-(p)SCC-co-culture, but not in mono-culture. These aforementioned changes in protein and gene expression strongly point towards important adverse biological consequences that may arise in case of the *in vivo* co-presence of ADSCs and SCCs.

In co-culture the gene expression or protein synthesis of a couple of pro-angiogenic proteins, such as FGF-2, IL-8, VEGF, CCL2 and CXCL6, is strongly up-regulated in ADSCs and/or SCCs. They are all known to support neoangiogenesis by increased migration and mitosis of endothelial cells, formation of new capillaries and vessel fenestrations or inhibition of endothelial cell apoptosis [15-26]. By supplying nutrition, this is an important component of tumor growth. Fittingly, we found an increased angiogenic potential in ADSC-A431-SCC-co-culture compared to respective mono-cultures *in vitro*. Several of these angiogenic proteins lead to an enhanced secretion of metalloproteinases and an increased MMP-activity, which not only facilitates neoangiogenesis, but also degradation of extracellular matrix and thereby invasive tumor growth. In contrast to that, the angiostatic CXCL-9 and CXCL10 [27] are down-regulated in co-culture or only expressed at a very low level.

IL-6 gene expression is highly up-regulated in ADSCs co-cultured with A431- and primary SCCs and the IL-6 protein level significantly increased in the co-culture medium, too. IL-6 has been reported to be a proliferative factor for diverse tumor types *in vivo*[28-30], and elevated serum levels of IL-6 have been associated with key features of malignancy, cancer progression and a poor clinical outcome in different types of cancers [31-37]. Presumably, one part of these IL-6 effects is achieved by increasing TWIST-expression, as well as stabilizing TWIST and inhibiting its degradation, thereby increasing cell motility and tumor progression [32]. TWIST1 encodes for the TWIST-related protein 1, a transcription factor, which acts as an oncogene in several cancers and has been shown to be involved in the development of resistance towards chemotherapeutic drugs and evading apoptosis [38]. When co-cultured with ADSCs TWIST1 was also significantly up-regulated in A431-SCCs cells in our experiment. TWIST1 plays a role in metastasis, presumably through up-regulation of MMP-expression and inhibition of tissue inhibitor of metalloproteinases-1 expression [39,40]. Indeed, the gene expression or protein synthesis of matrix metalloproteinases, such as MMP-1, −2, −3 or −7, was highly up-regulated when ADSCs and A431- or primary SCCs were co-cultured. In fact, MMP-7 was solely produced in co-culture but not in mono-culture. MMPs contribute to the breakdown of extracellular matrix in a multitude of physiological and pathological processes (for example, connective tissue remodeling, wound repair and metastasis). We could show a corresponding significant increase in the invasive behavior of primary SCCs and A431-SCCs and ADSCs, in the co-culture. Altered MMP expression has already been linked with poor disease prognosis in different human cancers and enhanced cancer cell invasion [41,42]. In addition to that, the CTKS gene expression was up-regulated in co-cultured A431-SCCs. CTKS encodes for cathepsin K, a cysteine protease with strong collagenolytic and elastolytic properties which is involved in extracellular matrix turnover [43,44]. CTKS up-regulation has been associated with tumor progression in squamous cell carcinomas of the skin [43].

Regarding the extracellular matrix (ECM), the expression of the fibronectin 1 gene *FN1* was slightly up-regulated in co-cultured A431-SCCs. High *FN1* expression or up-regulation in tumor cells has been associated with radioresistance, tumor progression and metastatic outgrowth [45-47]. Presumably, together with other extracellular matrix proteins, fibronectin forms a complex network around the tumor cells, regulating cell adhesion, migration and proliferation of tumor cells, fibroblasts and endothelial cells [48,49]. Integrins can bind fibronectin and are crucial in different tumor-associated processes, such as tumor cell growth, angiogenesis and metastasis. Co-culture strongly up-regulated the ITGA2B (integrin alpha 2 beta)-expression in ADSCs and *ITGB3* (integrin beta 3) gene expression in A431-SCCs. Increased *ITGB3* expression has been linked to an increase in migration and invasion, as well as a more aggressive phenotype of tumor cells, a progressed tumor grade and a poor prognosis [50,51].

Furthermore, ADSC-SCC-co-culture resulted in a very strong up-regulation of the gene expression of CSF-2 and −3 which resulted in a corresponding extraordinary increase in G-CSF and a moderate increase in GM-CSF concentration in the co-culture media. In general, G-CSF is associated with the stimulation of granulocytes and the proliferation of immature hematopoietic precursor cells during inflammatory responses. Tumor-derived G-CSF, however, has been demonstrated to be able to enhance tumor growth by inducing myeloid-derived suppressor cells, which suppress innate and adaptive immunity [52]. Thus, the increase in G-CSF by co-culturing ADSCs with SCCs might be associated with an increased tumor growth *in vivo*. Enhanced GM-CSF-levels in tumors are associated with a reduced cell proliferation but an increased migratory capacity, an increased tumor cell invasion by elevating MMP expression, and a higher vessel density [53]. This might be an explanation for the significantly reduced proliferation in co-cultured primary SCCs in our experiment.

Cancers often arise in association with a long lasting chronic inflammation [54]. High expression of some interleukins, such as IL-1α and IL-1β, has been associated with a more aggressive tumor type and poor prognosis [55-57]. ADSCs-SCCs-co-culture - both, with A431-SCCs and primary SCCs, led to a strong increase in IL-1α and β gene expression in ADSCs. IL-1β could only be detected in co-culture and not in mono-culture of ADSCs or primary SCCs. Both proteins, IL-1α and -β, have been implicated in tumor progression by inducing the expression of angiogenic and metastatic genes, cytokines and growth factors, such as MMPs, VEGF, IL-6 and 8, CCl2, CCL7, TNFα and TGFβ [58-60]. Importantly, IL1-β also stimulates an increase in prostaglandin E2 production through cyclooxygenase-2 (COX-2) induction. The highly up-regulated *PTGS2* gene in co-cultured ADSCs and also in A431-SCCs, encoding for COX-2, further supports this. All essential steps of malignant tumor progression, such as mutagenesis, mitogenesis, angiogenesis, reduced apoptosis, metastasis and immunosuppression, are associated with prostaglandin 2 [61]. Furthermore, it has been shown that infiltrates of immune cells and expression of inflammatory mediators, for example, cytokines and chemokines, are an important part of the tumor milieu and substantially contribute to tumor development and progression [62]. In this context, it is important to see that there is a robust up-regulation of different cytokines and chemokines, for example, CCL2, CCL3, CCL4, CCL5, CCL7, CCL8, CCL13, CCL20, CCL28, CXCL1, CXCL2, CXCL3, CXL5, CXCL6, CXCL11, CXCL12 and CXCL14 in ADSCs co-cultured with primary or A431-SCCs. CXCL9 expression can only be detected in co-culture. In tumors these chemokines attract special leukocyte/monocyte subpopulations - for example, tumor associated macrophages - which can promote tumor growth and metastasis by stimulating angiogenesis, ECM-degradation and tumor cell proliferation [63-66]. High numbers of these macrophages present at the tumor site have been associated with poor prognosis and disease progression [67]. CCL2 additionally promotes epithelial to mesenchymal transition and is linked to cancer cell migration and cancer progression [65,68]. In line with that, E-Cadherin was strongly down-regulated and N-Cadherin strongly up-regulated in co-cultured A431-SCCs, suggesting an epithelial-mesenchymal transition. E-Cadherin is an important cell-cell adhesion molecule in epithelial cells. Its down-regulation has not only been implicated in epithelial-mesenchymal transition, but also in an increase in cellular motility, invasion and metastasis. There is an inverse correlation among E-cadherin levels, tumor grade and patient mortality rates [69].

Different C-C motif ligand-chemokines (CCL) and C-X-C motif ligand chemokines (CXCL), such as CCL2, CCL7, CXCL1 and CXCL6, are also potent stimulators of pro-malignant features, such as tumor cell proliferation, migration and invasion, as well as angiogenesis. They promote tumor growth, facilitate metastasis, and high serum levels are associated with a poor outcome [60,66,70-73]. However, it is important to say that posttranscriptional modifications are common for many chemokines, affecting their biological function [74]. Thus, the up-regulation of their gene expression does not necessarily lead to a one-to-one change in protein activity and *in vivo* function.

In addition, *HGF* gene expression was strongly increased in co-cultured A431-SCCs, and also primary SCCs were exposed to a highly increased hepatocyte growth factor (HGF) level in co-culture compared to monoculture, due to high expression of HGF by ADSCs. HGF is known to be a potent angiogenic factor and to be associated with a more advanced tumor stage in different malignancies *in vivo*[75]. Furthermore, it has been demonstrated that HGF significantly increases the migration and invasion of esophageal SCCs *in vitro*, and that it is a major component of tumor progression induced by tumor-associated fibroblasts [76,77]. Thus, an increased HGF level in the ADSC-SCCs microenvironment, as shown in our experiment, might also promote tumor progression in an *in vivo* setting.

Other important genes were also up-regulated in co-cultured ADSCs: *LYPD3*, *WISP-1*, *TLR-2*, and *SERPINE-1. LYPD3*, encoding for the Ly6/PLAUR domain-containing membrane protein 3, has been shown to be down-regulated upon transition to dysplasia and carcinoma *in situ*, and being up-regulated again at the invasive front and in local lymph node metastasis of esophageal squamous cell carcinomas [78,79]. The *WISP-1* gene encodes the Wnt-induced secreted protein-1, which has been shown to enhance tumor cell migration by increasing MMP-2 expression, and to inhibit anti-tumor immunity by blocking the response of immune cells to IL-12. Furthermore, it has been associated with a poor prognosis in certain tumor types [80-82]. In addition, also, the TLR-2 (Toll-like receptor-2) has been associated with increased tumor progression and metastasis, as well as tumor angiogenesis [83].

SERPINE-1 encodes for the plasminogen activator inhibitor-1 (PAI-1) which is thought to facilitate tumor invasion by controlling the peritumor proteolytic microenvironment, regulating cell adhesion and migration, and stabilizing early capillary vessel structures. Elevated PAI-1 levels have been associated with a poor prognosis and a reduced disease-free survival in various malignancies [84,85].

Two other genes were also up-regulated in ADSCs co-cultured with A431 or pSCCs, respectively, *NR4A3* and *KISS1R. NR4A3* encodes for the orphan nuclear receptor *NOR-1*, an early immediate response gene, which among others is involved in cell growth and survival, apoptosis, glucose and lipid metabolism, and inflammation [86-88]. *KISS-1R* encodes for the KiSS1-derived peptide receptor, a G-protein-coupled receptor which is known to play multiple roles in cancer development and metastasis [89]. For both proteins, their distinct functions are discussed controversially in different tumor types.

The expression of the *CCND2* gene, encoding for cyclin D2, was up-regulated in primary SCCs co-cultured with ADSCs. Cyclin D2 plays an important role in the cell cycle at the transition from G1- to S-phase. High expression of *CCND2 in vitro* has been strongly associated with a more invasive SCC cell type *in vivo* and malignant progression [90,91].

Besides that, the primary SCCs gene expression did not seem to be much altered by co-culturing with ADSCs. Additionally, if cultured on the plate of the transwell system, co-culture of ADSCs and pSCCs significantly lowered the proliferation of both cell types. However, this should be interpreted cautiously, as it is not a sign of lack of interaction between or a hint towards a safe co-existence of these two cell types. The reduced cellular proliferation of primary SCCs determined under certain *in vitro* culture conditions might be a result of the increased GM-CSF-levels [53], a temporary effect or reflect a lack of supplemented nutrients in the *in vitro* situation. A risk of increased malignant features of SCCs in close vicinity of ADSCs is already suggested by the fact that the tumor cells are exposed to a new or more potent cocktail of cytokines, growth factors and matrix degrading enzymes secreted by the ADSCs. In line with that, we could show that the co-culture with ADSCs significantly increased the migration and invasion of primary SCCs *in vitro*.

The present study is first to show numerous interactions of ADSCs and a well-known and often used SCC cell line as well as primary SCCs *in vitro*. The results intensely point to clinically relevant consequences, such as an increase in tumor growth and earlier or more disseminated metastasis. Our results strongly support the hypothesis that there is an interaction between ADSCs and SCCs with potentially detrimental consequences for patients. To clarify the, in part, contradictory results and to get the necessary further insight into the far more complex situation *in vivo*, we are currently performing *in vivo* experiments in a rodent model. Nonetheless, our current results already need to fuel a necessary discussion about the safety of ADSC-based therapies, particularly in the skin.

## Conclusion

In summary, ADSCs significantly affect the multiple malignant properties of SCCs, such as invasion, gene expression and protein synthesis *in vitro*. Thereby ADSCs may strongly increase the risk of squamous cell carcinoma tumor growth and metastasis *in vivo*. Therefore, it is crucial to rigorously screen all patients for pre-malignant lesions prior to the injection of fat, stem cell-augmented fat or isolated ADSCs in the skin or adjacent tissues to avoid a potential co-localization of ADSCs and SCCs. Informed consent of patients to such procedures will need to include the explanation of an increased risk of developing a malignant skin condition or the faster growth and dissemination of a possibly pre-existing skin cancer.

## Abbreviations

ADSCs: Adipose tissue-derived stem cells; CCL: C-C motif ligand-chemokines; CEACAM-1: Carcinoembryonic antigen cell adhesion molecule; COX-2: Cyclooxygenase-2; CT: Cycle threshold; CXCL: C-X-C motif ligand chemokines; ECM: Extracellular matrix; EMT: Epithelial-mesenchymal-transition; EPCAM: Epithelial cell adhesion molecule; HGF: Hepatocyte growth factor; HUVEC: Human umbilical vein endothelial cells; IL: Interleukin; ITGB3: Integrin beta 3; MMPs: Matrix metalloproteinases; PMA: Phorbol 12-myristate 13-acetate; pSCCs: primary squamous cell carcinoma cells; SCCs: Squamous cell carcinoma cells; SD: Standard deviation; TLR-2: Toll-like receptor-2.

## Competing interests

The authors declare that they have no competing interests.

## Authors’ contributions

EK conceived of the study, coordinated the study, performed the statistical analysis and drafted the manuscript. FG drafted the manuscript, and helped in performing the statistical analysis and the interpretation of the data. FP carried out the flow cytometry and cell differentiation. UL isolated and cultured the cells, and participated in the analysis of gene expression. GG conceived of the study. VD carried out the gene expression analysis and the protein measurements. All authors read and approved the final version of the manuscript.

## Supplementary Material

Additional file 1: Table S1 A) Co-culture of ADSCs and A431-SCC-cell line - Part 1. Minor changes in the gene expression of ADSCs - Part 2. Minor changes in the gene expression of A431-SCCs. B) Co-culture of ADSCs and primary SCCs. Part 1. Minor changes in the gene expression of ADSCs. Part 2. Changes in the gene expression of primary SCCs. Table S1 Part A. Minor changes in the gene expression levels of ADSCs and A431-SCCs in co-culture compared to mono-culture. GUSB was used as referring housekeeping-gene. Only changes of 2.5-fold or higher are displayed. Part 1 displays the changes in the gene expression levels of ADSCs. Part 2 shows the changes in the gene expression levels of A431-SCCs. Arrows mark an up- (↑) or down-regulation (↓) of the gene expression compared to the referring mono-culture. Table S1 Part B. Minor changes in the gene expression levels of ADSCs and pSCCs in co-culture compared to mono-culture. GUSB was used as referring housekeeping-gene. Only changes of 2.5-fold or higher are displayed. Part 1 displays the changes in the gene expression levels of ADSCs. Part 2 shows the changes in the gene expression levels of pSCCs. Arrows mark an up- (↑) or down-regulation (↓) of the gene expression compared to the referring mono-culture. Click here for file
