# Supplementary material for: Alterations of gene expression and protein synthesis in co-cultured adipose tissue-derived stem cells and squamous cell-carcinoma cells: consequences for clinical applications
Source: Stem Cell Res Ther. 2014 May 12;5(3):65. doi: 10.1186/scrt454 (PMC4076640; doi:10.1186/scrt454)
Supplement: Additional file 1: Table S1 — A) Co-culture of ADSCs and A431-SCC-cell line - Part 1. Minor changes in the gene expression of ADSCs - Part 2. Minor changes in the gene expression of A431-SCCs. B) Co-culture of ADSCs and primary SCCs. Part 1. Minor changes in the gene expression of ADSCs. Part 2. Changes in the gene expression of primary SCCs. Table S1 Part A. Minor changes in the gene expression levels of ADSCs and A431-SCCs in co-culture compared to mono-culture. GUSB was used as referring housekeeping-gene. Only changes of 2.5-fold or higher are displayed. Part 1 displays the changes in the gene expression levels of ADSCs. Part 2 shows the changes in the gene expression levels of A431-SCCs. Arrows mark an up- (↑) or down-regulation (↓) of the gene expression compared to the referring mono-culture. Table S1 Part B. Minor changes in the gene expression levels of ADSCs and pSCCs in co-culture compared to mono-culture. GUSB was used as referring housekeeping-gene. Only changes of 2.5-fold or higher are displayed. Part 1 displays the changes in the gene expression levels of ADSCs. Part 2 shows the changes in the gene expression levels of pSCCs. Arrows mark an up- (↑) or down-regulation (↓) of the gene expression compared to the referring mono-culture. [file scrt454-S1.docx]

**Additional table 1. A Co-culture of ADSCs and A431-SCC-cell line**

***Part 1. Minor changes in the gene expression of ADSCs***

| **gene** | fold **ADSC** mono-/co-culture | up- / down-regulation |
| --- | --- | --- |
| **CCND1** | **2.7** (0.1) | **↓** |
| **CCND3** | **2.7** (0.1) | **↓** |
| **CYCS** | **2.7** (0.1) | **↓** |
| **DVL1** | **2.7** (0.1) | **↓** |
| **E2F1** | **3.2** (0.2) | **↓** |
| **FGF-2** | **2.5** (1.3) | **↑** |
| **FGFR4** | **5.4** (1.9) | **↑** |
| **MCAM** | **4.0** (2.7) | **↑** |
| **MMP-2** | **3.4** (1.0) | **↑** |
| **NFKBIA** | **4** (0.0) | **↑** |
| **NFKB2** | **2.7** (0.9) | **↑** |
| **PIK3CD** | **3.3** (0.9) | **↑** |
| **PPBP** | **3.3** (1.6) | **↑** |
| **RELB** | **2.7** (0.9) | **↑** |
| **SPP1** | **2.6** (0,1) | **↓** |
| **STAT4** | **4.0** (0.0) | **↑** |
| **TYMP** | **3.4** (0.9) | **↑** |

***Part 2. Minor changes in the gene expression of A431-SCCs***

| **gene** | fold **A431-SCC** mono-/co-culture | up- / down-regulation |
| --- | --- | --- |
| **CYCS** | **3.0** (0.1) | **↓** |
| **ETV4** | **2.5** (1.4) | **↑** |
| **FOS** | **4.0** (0.0) | **↓** |
| **IKBKE** | **2.5** (0.1) | **↓** |
| **MCAM** | **2.3** (1.3) | **↑** |
| **PMAIP1** | **3.1** (0.1) | **↓** |
| **PPBP** | **2.5** (1.5) | **↑** |

**Additional Table 1 Part A. Minor changes in the gene expression levels of ADSCs and A431-SCCs in co-culture compared to mono-culture.** GUSB was used as referring housekeeping-gene. Only changes of 2.5-fold or higher are displayed. Part 1 displays the changes in the gene expression levels of ADSCs. Part 2 shows the changes in the gene expression levels of A431-SCCs. Arrows mark an up- (↑) or down-regulation (↓) of the gene expression compared to the referring mono-culture.

**Additional table 1. B Co-culture of ADSCs and primary SCCs**

***Part 1. Minor changes in the gene expression of ADSCs***

| **gene** | fold **ADSC** mono-/co-culture | up- / down-regulation |
| --- | --- | --- |
| **BCL2** | **3 (0.1)** | **↓** |
| **CCL2** | **4.0 (0.0)** | **↑** |
| **CCL3** | **2.7 (0.9)** | **↑** |
| **CCL7** | **3.4 (1.0)** | **↑** |
| **CCL8** | **3.4 (0,9)** | **↑** |
| **CCL13** | **2.7 (0.9)** | **↑** |
| **CXCL11** | **2.7 (1.0)** | **↑** |
| **CXCL12** | **2.7 (1.9)** | **↑** |
| **CXCR4** | **3.1 (1.1)** | **↑** |
| **FGFR4** | **2.7 (1.0)** | **↑** |
| **FOS** | **3.0 (0.8)** | **↓** |
| **MMP-2** | **2.7 (1.0)** | **↑** |
| **MMP-9** | **2.7 (1.0)** | **↑** |
| **PIK3CD** | **3.4 (0,9)** | **↑** |
| **TPBG** | **2.7 (1.0)** | **↑** |

***Part 2. Changes in the gene expression of primary SCCs***

| **gene** | fold **primary** **SCC** mono-/co-culture | up- / down-regulation |
| --- | --- | --- |
| **CCL5** | **3.0 (0.1)** | **↓** |
| **TNF** | **2.7 (0.1)** | **↓** |

**Additional Table 1 Part B. Minor changes in the gene expression levels of ADSCs and pSCCs in co-culture compared to mono-culture.** GUSB was used as referring housekeeping-gene. Only changes of 2.5-fold or higher are displayed. Part 1 displays the changes in the gene expression levels of ADSCs. Part 2 shows the changes in the gene expression levels of pSCCs. Arrows mark an up- (↑) or down-regulation (↓) of the gene expression compared to the referring mono-culture.
